# An integrated cognitive framework for understanding modern cities

**DOI:** 10.1007/s43762-022-00065-8

**Published:** 2022-10-07

**Authors:** Renzhong Guo, Wuyang Hong, Biao He, Weixi Wang, Xiaoming Li, Minmin Li, Lin Jiang

**Affiliations:** 1 Research Institute for Smart Cities, School of Architecture and Urban Planning, Shenzhen, 518060 China; 2 Technology Innovation Center for Territory Spatial Big-Data, Ministry of Natural Resources, Shenzhen, 518060 China; 3 Key Laboratory of Urban Land Resources Monitoring and Simulation, Ministry of Natural Resources, Shenzhen, 518060 China

**Keywords:** Modern city, Characteristic recognition, Integrated framework, Theoretical system, Governance system

## Abstract

Modern urban development urgently requires a new management concept and operational mechanism to encourage the exploration of frameworks for cognizing and studying urban characteristics. In the present study, modern cities are first understood from the perspective of their basic theoretical evolution. Each modern city is seen as a complex system of organic life forms. Urban information science propels modern urban research in the direction of rationality. This paper also presents the new characteristics of modern cities (and how they have changed) in relation to external structure and internal functions. It examines the generation of urban problems and governance adaptability. On this basis, this paper proposes a cognitive model for studying modern cities, integrating basic theoretical, methodological support, and governance systems. It discusses the basic rationale and core idea for constructing each of these three systems. The research aims to guide and implement modern urban construction and sustainable development in a more effective way.

## Introduction

Throughout the history of urban research, city evolution has always reflected current development-stage characteristics and changed the urban-science research paradigm. Traditional cities are seen as collections of locations. The proposed theory of “garden cities” has marked the initial formation of an urban-research system, from the perspective of “regional science.” Societal needs and progress in urban technology has led to the emergence of classical regional-science theories (e.g., the “growth pole,” “central place,” and “spatial structure” theories), enabling the use of various types of research thinking (e.g., linearity, symmetry, reductionism, and the equilibrium view) to analyze the state and operational mechanism of cities (Allen, 2012; Shi et al., 2021, b). As new information, communication technology, and urbanization emerge, the concept of “the city” continues to expand, while the triadic space formed by linking geographic, social, and information spaces reshapes its structure and functional layout (Guo et al., 2020). The composition and organization of elements, the frequency and mode of interaction, and the scale and levels of activity space within urban systems have become increasingly complex. The cities we are familiar with have quietly transformed, while urban diseases and planning and management problems have emerged in parallel. Urban science now faces multitudinous challenges, including new theories and technologies (Bibri, 2021; Hong et al., 2022).

The world has entered the urban age. Worldwide, the vast majority of people are expected to live in cities by the end of the twenty-first century (Batty, 2013). Technological changes and the growth of social needs have driven urban development toward a new phase of digitalization, informatization, and intellectualization (Cocchia, 2014). Modern cities urgently need complementary theoretical, methodological, and practical frameworks. Focusing on their development and theoretical evolution, this study elaborates the main characteristics and existing problems of modern cities. It proposes theoretical, methodological, and governance systems for studying modern cities; presents the fundamental rationale and core concept for constructing each system; and establishes a three-dimensional (3D) integrated framework for cognizing modern cities to meet the requirements of sustainable urban development in the new era.

## A basic understanding of modern cities

Modern cities are a new urban form, on par with ancient and early-modern cities. Urban research has passed through three stages, evolving from central-location theory to the early-modern central paradox, and the modern theory of complex systems. Location theories, represented by von Thünen’s (1826) “agricultural location theory,” Webber’s (1909) “industrial location theory,” and Christaller’s (1933) “urban location theory,” started a quantitative geographical revolution (Liu et al., 2014). Later, Edward Glaeser and other urban-research scholars summarized the theoretical development of early modern cities and formulated the “paradox of the modern metropolis.” In the 1980s, scholars from the Santa Fe Institute in the United States and University College, London in the United Kingdom, argued that urban-science theory was a complex science of constantly evolving, nonlinear, and non-equilibrium systems. This marked the beginning of the system-theory stage of urban theory (Batty, 2007).

Conceptually, a modern city is a complex system of organic life forms. Early urban-research scholars defined the city as an economic organization, an institutional process, and a place for social behavior (Howard, 1946). Cities nurture art and are themselves art; they create theater and are themselves theater (Batty, 2018). Scholars of the new urban scientism, represented by Fuller and Moore (2017), believed that it was not enough to treat cities simply as spaces and places, or to study the location of various city elements. Instead of analyzing city elements in isolation, they had to rethink the problem of location overall, while focusing on the network of relationships formed by people who assembled in cities. It can be argued that early urban-research scholars regarded cities as static “machines,” while the new urban-research scholars see cities as living “organisms.” The shift from “machines” to “organisms” is the best way to describe the shift in thinking about cities (Batty, 2012).

Technologically, urban information science drives modern urban research in the direction of rationality. Early urban research emphasized the fields of historical and economic geography. Later, the focus shifted to urban, demographic, and social geography, engaging with more scientific fields, including economics, planning, and management. Since the beginning of the twenty-first century, information-technology tools have opened a window for cognizing and understanding cities from a data-science perspective. An emerging field of knowledge, focused on social and industrial needs, aims to establish a science that uses data and information technology to explain urban growth, sprawl, and decline. Research methods that apply big data and quantitative analyses as primary tools have improved our ability to analyze and understand complex urban systems. Such methods are driving urban research to develop in a more rational and scientific manner. Australian scholars, including Foth et al. (2011), have established an urban informatics-research laboratory and attempted to define “urban information science,” a field that has been further expanded and supplemented by later scholars. Bettencourt and West (2010) analyzed urban problems from the perspective of physicists and physics research, empirically deriving universal urban patterns from big data and mathematical models. These demonstrate the essential role played by urban information science in achieving a scientific understanding of the impact of cities on society and the environment. Through extensive empirical research, urban-research scholars, represented by Batty (2013), have shown that complex cities can be represented using simple rules.

## The main characteristics of modern cities

**Fig. 1:**
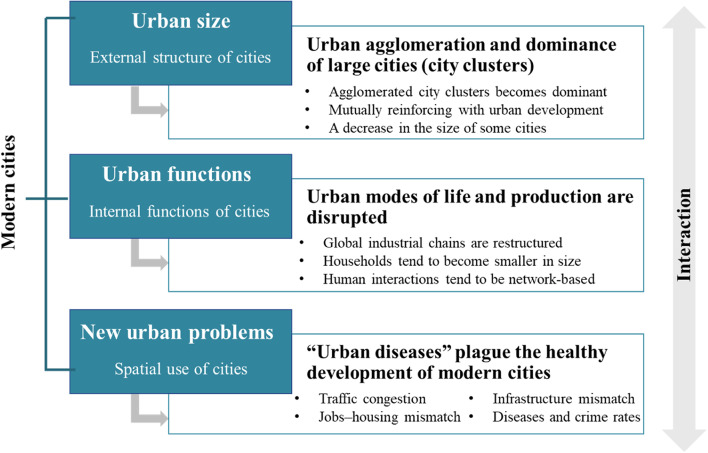
The key characteristic components of modern cities

To explore and develop a framework for studying modern cities, researchers must analyze their spatial structures and internal spaces, identifying key problems associated with human needs and urban development (Fig. 1). Overall, the agglomeration and dominance of large cities (city clusters) have become the main trend in modern urban development. Urban spaces display complex characteristics, including vertical expansion, multicenter cooperation, and extravisual growth (e.g., intensive and efficient production spaces and a functional, clustered, and 3D layout). By contrast, living spaces have decreased in size and become network-based and virtual. New urban problems, including traffic congestion, the mismatch between jobs, housing, and infrastructure, and urban safety, have also arisen.

### Urban agglomerations and large-city dominance (city clusters)

Urban agglomerations and large-city dominance (city clusters) have become the main trend in modern urban development. In 1900, urban populations accounted for 16.40% of the world’s total population. In 2007, urban populations exceeded rural populations for the first time. By 2050, 66% of people are expected to live in cities (Camero & Alba, 2019; United Nations P D, 2016). As large cities provide more opportunities and resources, migration to urban areas has become a universal pattern (Khanna, 2016). Since the 1950s, cities have experienced explosive growth in size and population (Zhao et al., 2017), evolving from world cities in the 1980s and global cities in the 1990s to megacities in the twenty-first century. Cities that used to be hundreds of kilometers apart have merged to form huge city clusters.

The size of a city is closely related to its development (Parkinson et al., 2015). The larger the city, the more favorable its output, and the more of its infrastructure input can be saved. The metrics of a city (e.g., economic income, gross domestic product (GDP), and patents) are superlinearly related to population size, with a scaling factor close to 1.15. Input into the infrastructure of a city (e.g., gas stations, roads, water, electricity, and gas lines) is sublinearly related to size, with a scaling factor close to 0.85 (Bettencourt & West, 2010). In addition, a larger city can accelerate technological innovation, improve industrial chains, and increase productivity more quickly than a small city. These factors further promote urban agglomeration, which enhances interpersonal communication and contact, while accelerating the generation of new technologies, the upgrading of industrial chains, and the development of Internet-based industries (Pentland, 2015; Zhao et al., 2017).

At the same time, researchers are increasingly focusing on negative factors affecting urban agglomeration, such as high housing prices, exposure to epidemic risks, and immutable living conditions. The negative effects brought by urban agglomeration should also be paid attention to. Urban agglomeration has increased the size of some cities while shrinking others (Dong & Li, 2020; Martinez-Fernandez et al., 2012). The pattern in which sharp increases and decreases in the urban population coexist differs from conventional population migration (e.g., from rural to urban areas during the Industrial Revolution). Instead, the modern pattern involves the movement of people from one city to another, and specifically from small to large cities and vice versa. Shrinking cities are primarily found in Europe, the United States, and the Asian countries of Japan and China (Yang & Pan, 2020). Over the past five decades, approximately 370 cities worldwide, with populations over 100,000, have lost more than 10% of residents, due to outmigration (Long & Gao, 2019).

### Systematic changes in urban modes of life and production

Global industrial chains accelerate more quickly during urbanization. Periodic international financial crises hasten the rebalancing of the global economy, optimizing and changing the global governance system. They also cause a continuous adjustment of the world’s industrial structures, promote international industrial transfers, and lead to a refined and specialized division of labor in industrial chains. The rise of information technology, particularly the Internet, and the emergence of new technologies (e.g., big data, cloud computing, and artificial intelligence) have caused the industrial structure to accelerate more quickly. The boom in the sharing and digital economies and strategic emerging industries has gradually changed internal-structure divisions in the traditional tertiary sector of the economy. This context has increased the mobility of enterprise employees and decreased their stability, while gradually shortening the lifespan of enterprises. The average tenure of employees at ten well-known global companies, including Facebook, Google, and Oracle, is less than two years. The average lifespan of the world’s top 500 companies decreased from 60 years in the 1950s to less than 20 years in 2017.

Urbanization is also disrupting traditional family structures. Households have become smaller, decreasing in China decreased from 4.36 persons per household in 1982 to 2.62 persons per household in 2020. In Japan, the average household is even smaller: in 2015, single-person households accounted for more than 30% of all households in Japan. Most urban residents live in single-family households with core members. The number of three- or four-generation households continues to fall, making it increasingly difficult to provide supports for elderly people or educate children.

In addition, urbanization gradually changes the way in which people socialize (Ye & Liu, 2018). Socialization tends to be network-based. The residents of a large city live fast-paced lives and have complex social networks. In a joint study carried out by Facebook and the University of Milan, calculations based on the data of 721 million Facebook users (via accurate network algorithms) showed that every two users could be connected through 4.74 intermediaries, on average. Online social networking has created closer and more complex connections between people. Where the standard was once six degrees of separation, it is now under five.

### “Urban diseases” plague the healthy development of modern cities

Traffic congestion is a common problem in large cities worldwide; it is significantly positively correlated with the city size (Louf & Barthelemy, 2014). Currently, road building and navigation systems are the primary approaches used to solve urban traffic congestion. On average, vehicle mileage increases by 10% for every 10% increase in road capacity (Wang et al., 2016). The optimized solutions provided by navigation systems increase, rather than reduce, urban residents’ average commuting distance. Neither approach addresses the root cause of traffic congestion.

Mismatched infrastructure is another major problem associated with modern cities. Typically, the larger the city, the more severe the mismatch between jobs and housing. Unlike traditional modes of commuting (e.g., walking, bicycles, and horse-drawn carriages), automobiles and subways have increased urban commuting distances and the mismatch between jobs and housing, making it commonplace for urban residents to spend more time commuting. In the 10 Chinese cities with the highest GDP (e.g., Beijing, Shanghai, and Chongqing), residents commute an average distance of 10 km, travelling for more than 40 minutes. Long commutes cause residents to consume energy unnecessarily and increase carbon emissions in urban areas.

Disease and crime also represent major challenges to current urban development. In 2019, coronavirus disease (commonly known as COVID-19) wreaked havoc across the globe, particularly in relation to urban public health (Wang & Tang, 2020). Cities are places in which diseases such as HIV/AIDS and mental and psychological disorders are widely and easily transmitted. Crime rates rise as cities increase in size. Recent research has shown that large cities have considerably higher crime rates and higher levels of violence than small cities or rural areas (De Nadai et al., 2020). Moreover, urban problems, including the widening gap between rich and poor, increase urban pollution. Cases of urban waterlogging occur more frequently as cities expand.

## An integrated framework for cognizing modern cities

**Fig. 2:**
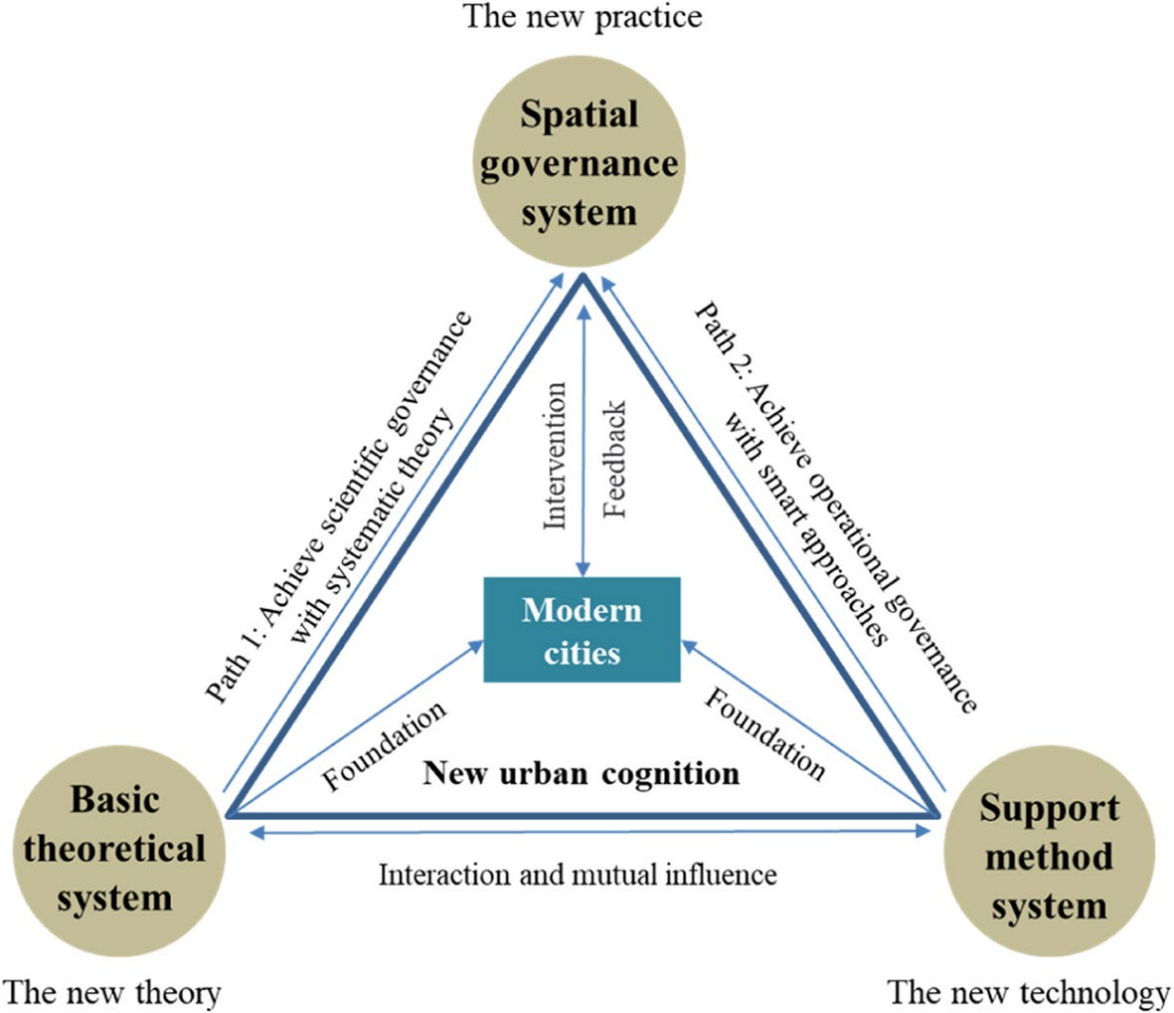
The cognitive and investigative framework for modern cities

The wave of rapid urbanization has led to a change in the connotative meaning of cities. Modern cities exhibit different urban characteristics, producing a series of new urban issues. This study therefore proposes a three-part framework for studying modern cities, incorporating the construction of a basic theoretical system, a methodological support system, and a spatial governance system for modern cities (see Fig. 2). Complex-system theory and methodology provide new opportunities to solve modern urban problems and break through bottlenecks in urban development (path 1). New-generation information technologies (e.g., cloud computing, big data, AI, the Internet of Things, and virtual reality) provide innovative drivers for realizing urban governance (path 2).

### Construction of a basic theoretical system for modern cities

A basic theoretical system can provide a fundamental vehicle for modern urban research. It is necessary to reexamine classical theories (e.g., location theory, urban planning principles, urban spatial structure, travel behavior, urban morphology, urban vitality, and urban design theories) and to evaluate their role and significance within the theoretical system of modern cities. To develop a “new” urban-theory system, it is essential to combine theories from various disciplines (e.g., social physics, management, psychology, data science, and geospatial informatics). Based on the premise that social networks are used extensively, modern social physics can study the mechanism through which social behavior is formed by observing single individuals and using “quantum mechanics-like” principles to derive patterns of social-group behavior (Pentland, 2014, 2015). Based on a combination of key theories from the fields of physics, management, and psychology, researchers can understand the formed urban characteristics of modern cities as a consequence of the coexistence and interaction of people, facilities, the environment, and services in urban spaces (Dunbar, 2010; Amabile et al., 2005; Boehm & Lyubomirsky, 2008; Chen et al., 2016).

**Fig. 3:**
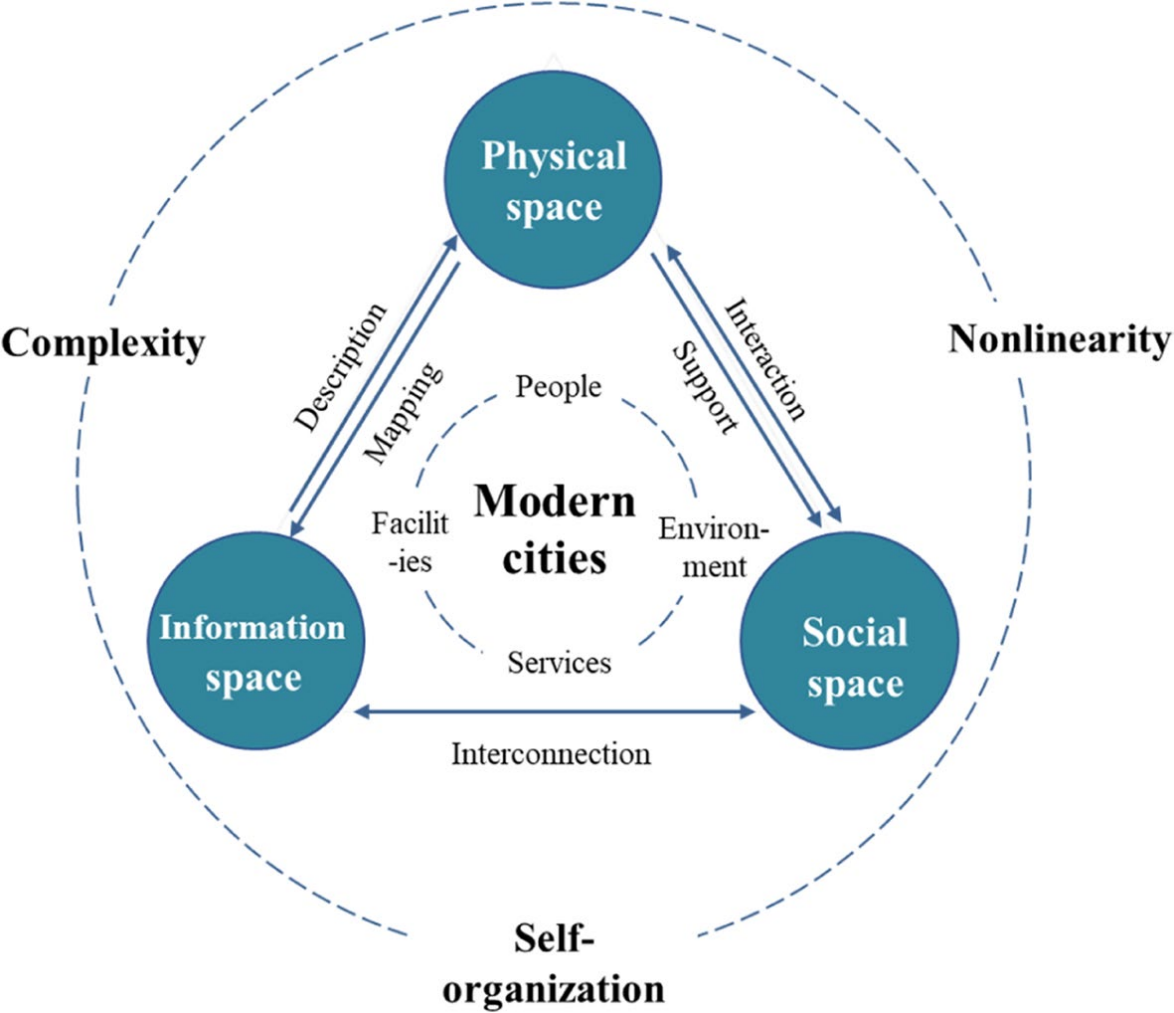
Basic theoretical system of modern cities

Integrated multidisciplinary theories can provide a more effective tool for explaining the characteristics of modern cities. This study uses the theory of complex urban systems, combined with multidisciplinary perspectives, to construct a basic theoretical system, in which the modern city is a complex organic system composed of geographic, information, and social spaces (see Fig. 3). The geographic space carries the basic physical elements of the city and constitutes the substrate for building an information space. The social space mainly comprises urban residents and their activities, interacting with geographic space. Overall, the physical city, at the geographic and social-space levels, is interwoven with the virtual city at the information-space level, resulting in a pattern of reality–virtuality, intermingled with urban development. Collectively, they form a modern city characterized by complexity, nonlinearity and self-organization.

### Construction of a support method system for modern cities

Based on their application characteristics in the era of big data, city data can be grouped into four categories: basic, statistical, multisource-spatiotemporal, and social-network data (Long, 2019). These categories provide important support for a comprehensive understanding and in-depth analysis of urban issues. With the emergence of new technologies (e.g., sensors, drones, wearable devices, and time-lapse photography), many new types of data (e.g., mobile phone signaling data, points of interest (POIs), location-based services (LBSs), street-view images, and social networks) are emerging in large quantities. New technological tools are needed to collect new data and develop new data-processing methods to re-interpret modern cities (Long, 2020). The application of new spatial-information technologies and methods has fostered the development of urban research, markedly enhancing the computing, sensing, transmission, and storage capabilities available for urban operations. It has also provided methodological guidance for establishing “new” cognitions and deepening “new” research on modern cities (Wei & Wang, 2020; Ye et al., 2021).

**Fig. 4:**
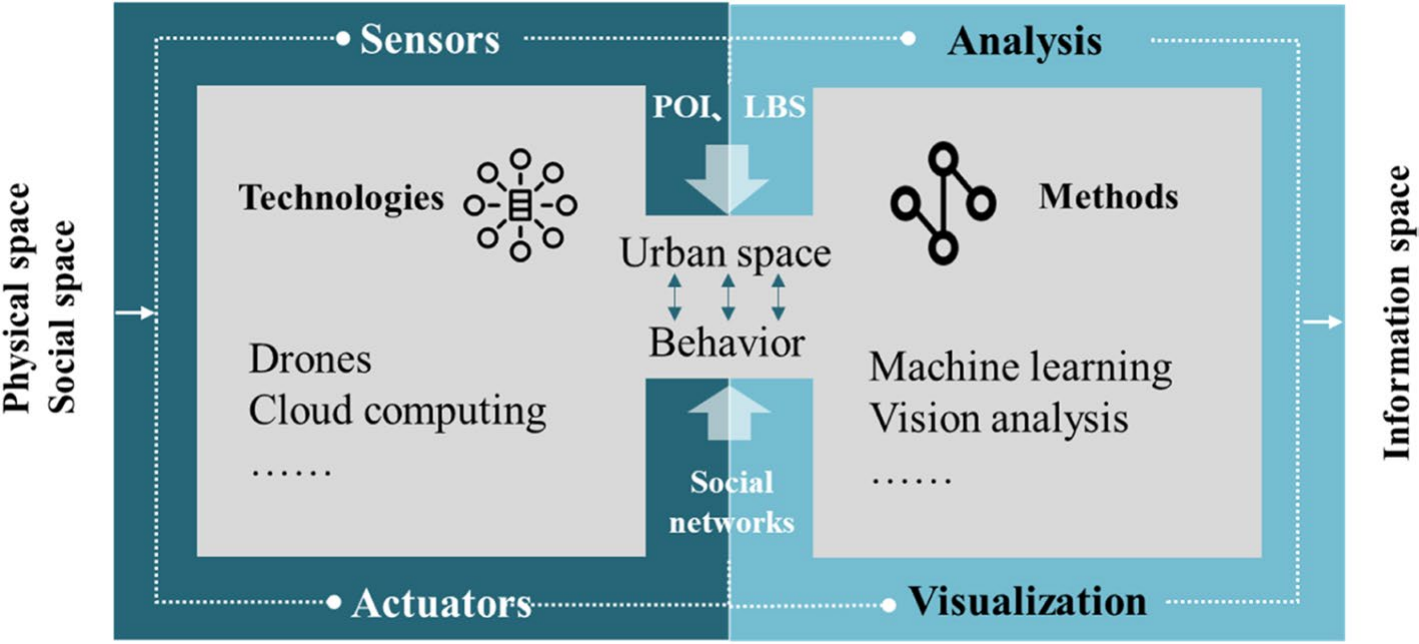
Methodological support system for modern cities

In this research, new data, technologies, and methods are used to construct a methodological support system for modern cities (see Fig. 4). A modern city comprises interwoven entity relations, potential rules, and patterns of operation, including physical and social spaces, formed from the interaction of individual behavior and urban spaces. Based on new devices (e.g., sensors, actuators, and drones), combined with new data (e.g., POIs, LBSs, mobile-phone signaling data, and social networks), new methods (e.g., big data analysis and visualization, computer-vision analysis, and machine learning) are used to realize multiple functions, including ubiquitous connectivity, virtuality–reality mapping, and real-time simulation; they also promote digital and intelligent development within the information space of a modern city. This methodological system enables researchers to conduct modern urban research in a more comprehensive and scientific way, while identifying the characteristics of a modern city via finer and richer details on multiple scales (e.g., spatial structure, urban morphology, and urban behavior).

### Construction of a spatial governance system for modern cities

Urban spatial governance is based predominantly on urban spatial elements, constituting an important component of urban governance (Wu, 2002). The emergence of various “new” urban problems in modern cities reflects the presence of numerous problems in current urban spatial governance, including the lack of closed-loop integrated management, difficulties in determining responsible subjects, lagging problem feedback mechanisms, the absence of long-term management mechanisms, and underdeveloped intelligent applications (Gabrys, 2014; McGuirk et al., 2021; Shi et al., 2021, b). The traditional models, which rely on administrative power or focus solely on social autonomy, cannot meet the current need for urban spatial governance. It is therefore crucial to form a spatial governance system for modern cities that draws on the collective participation of multiple subjects, based on new theories and technologies, to achieve appropriate interventions and timely feedback.

**Fig. 5:**
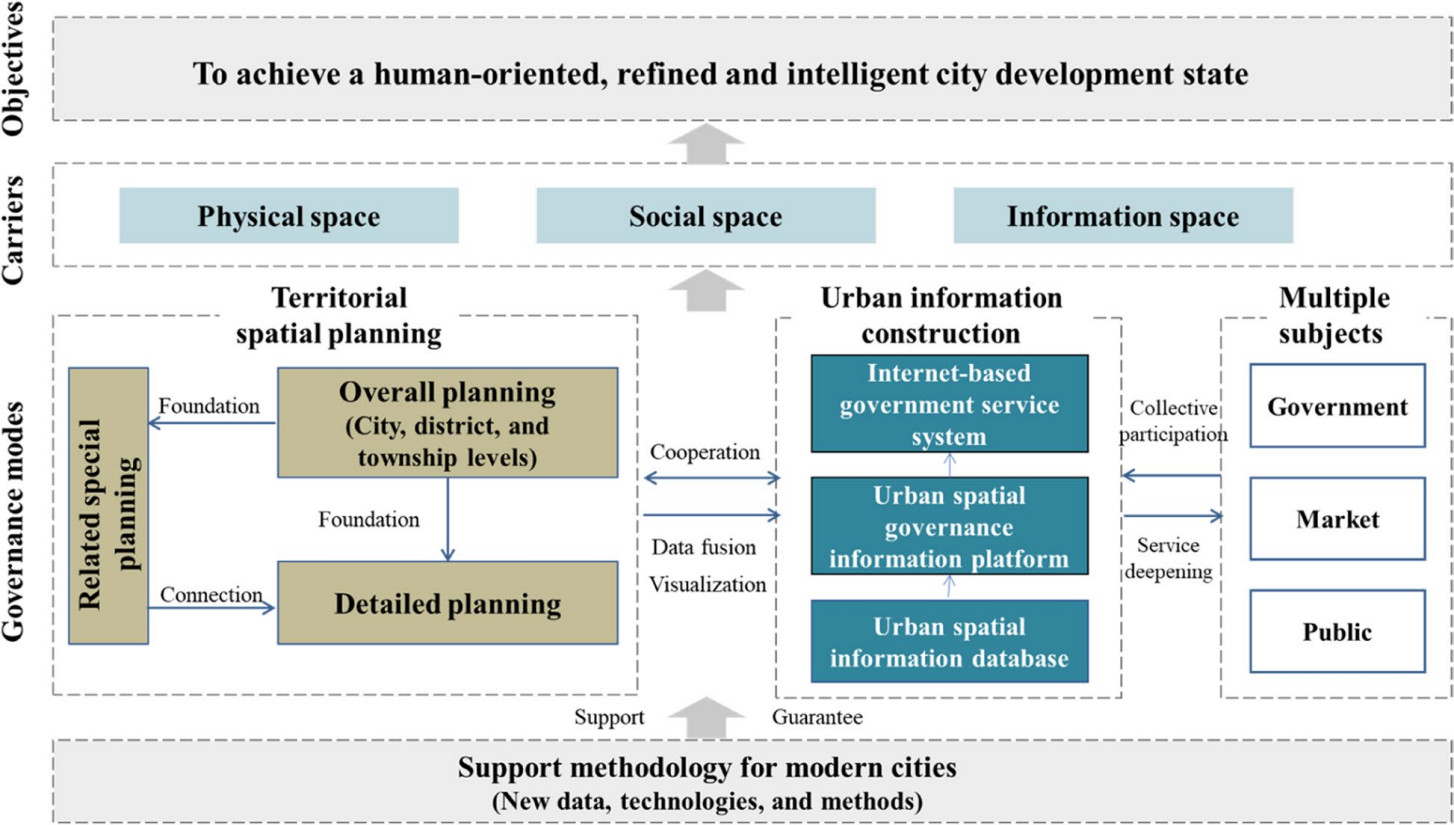
A spatial governance system for modern cities

There are two main ways to achieve humanistic, refined, and intelligent spatial governance in modern cities (see Fig. 5): (1) The basic theoretical system for understanding modern cities is used to form a systematic interpretation, ensuring scientific spatial governance. Currently, spatial governance in most traditional modern cities remains at the level of geographic space. To some extent, it neglects the important role played by information and social spaces. The spatial governance system for modern cities considers three types of urban spaces systematically, transforming and innovating urban spatial governance, based on territorial spatial planning, modern urban information construction, and the needs of multiple subjects. (2) Based on the support methodology for modern cities, combined with game-changing new technologies and methods, urban problems are simulated and predicted through a quantitative and refined analysis. This analysis interlinks and iteratively optimizes different urban spaces, allowing intelligent means to support and ensure scientific governance of modern cities. Humanistic, refined, and intelligent urban spatial governance can be achieved at the application level through cooperation between territorial spatial planning and established modern urban information systems (e.g., the urban spatial-information database, urban spatial-governance-information platform, and Internet-based government service system).

## Conclusions

After a long history of human social development, cities are gradually leaving behind their traditional inertia and modes of operation, giving rise to new development needs, including basic theories, support technologies, and governance models. Although cities are still called cities, the world has changed. Externally, transportation and communication technologies have developed; the functional links between cities have strengthened; and urban areas have developed multicenter and network-based characteristics. Within cities, individual behavior tends to be fragmented in terms of time and free in terms of activity locations. Urban spaces display complex characteristics, including vertical expansion, multicenter cooperation, and extravisual growth, leading to a series of devastating urban diseases. The traditional model, which relies on administrative power or focuses solely on social autonomy, cannot meet the real challenges facing urban governance. A new urban-research paradigm is urgently needed to guide and achieve modern urban construction and sustainable development more effectively.

Modern cities have evolved into complex systems, encompassing multiple scenarios that interact organically in geographic, information, and social spaces. Driving forces, based on new information technologies and intelligent urban construction, lead to coexisting opportunities and challenges in modern urban development. This study attempts to construct a framework for studying modern cities by integrating three systems: theoretical, methodological, and governance. This framework can be understood as a 3D structural model for addressing modern cities as a complex system. This study proposes a research direction that takes advantage of the rationality of “complex system theory” to enhance the rationality of urban planning and management decisions. Future studies will continue to strive to understand the characteristics of modern cities. The characteristics of typical cities (e.g., Shenzhen) will be identified and quantitatively analyzed based on their spatiotemporal big data to further improve the basic theoretical, support methodological, and spatial governance systems established in this study from an empirical perspective.

## Data Availability

Not applicable.
